# The influence of growth rate-controlling feeding strategy on the surfactin production in *Bacillus subtilis* bioreactor processes

**DOI:** 10.1186/s12934-024-02531-w

**Published:** 2024-09-30

**Authors:** Eric Hiller, Manuel Off, Alexander Hermann, Maliheh Vahidinasab, Elvio Henrique Benatto Perino, Lars Lilge, Rudolf Hausmann

**Affiliations:** Department of Bioprocess Engineering, Institute of Food Science and Biotechnology, University of Hohenheim, Stuttgart, Germany

**Keywords:** *Bacillus subtilis*, Surfactin, Bioreactor, Bioprocess engineering, Fed-batch

## Abstract

**Background:**

The production of surfactin, an extracellular accumulating lipopeptide produced by various *Bacillus* species, is a well-known representative of microbial biosurfactant. However, only limited information is available on the correlation between the growth rate of the production strain, such as *B. subtilis* BMV9, and surfactin production. To understand the correlation between biomass formation over time and surfactin production, the availability of glucose as carbon source was considered as main point. In fed-batch bioreactor processes, the *B. subtilis* BMV9 was used, a strain well-suited for high cell density fermentation. By adjusting the exponential feeding rates, the growth rate of the surfactin-producing strain, was controlled.

**Results:**

Using different growth rates in the range of 0.075 and 0.4 h^-1^, highest surfactin titres of 36 g/L were reached at 0.25 h^-1^ with production yields Y_P/S_ of 0.21 g/g and Y_P/X_ of 0.7 g/g, while growth rates lower than 0.2 h^-1^ resulted in insufficient and slowed biomass formation as well as surfactin production (Y_P/S_ of 0.11 g/g and Y_P/X_ of 0.47 g/g for 0.075 h^-1^). In contrast, feeding rates higher than 0.25 h^-1^ led to a stimulation of overflow metabolism, resulting in increased acetate formation of up to 3 g/L and an accumulation of glucose due to insufficient conversion, leading to production yields Y_P/S_ of 0.15 g/g and Y_P/X_ of 0.46 g/g for 0.4 h^-1^.

**Conclusions:**

Overall, the parameter of adjusting exponential feeding rates have an important impact on the *B. subtilis* productivity in terms of surfactin production in fed-batch bioreactor processes. A growth rate of 0.25 h^-1^ allowed the highest surfactin production yield, while the total conversion of substrate to biomass remained constant at the different growth rates.

**Supplementary Information:**

The online version contains supplementary material available at 10.1186/s12934-024-02531-w.

## Background

The species *Bacillus subtilis* is known for the biosynthesis of various secondary metabolites with bioactive properties [1]. One representative is surfactin, a lipopeptide consisting of a cyclic peptide moiety with seven amino acids (L-Glu, L-Leu, D-Leu, L-Val, L-Asp, D-Leu and L-Leu) and a β-hydroxy fatty acid chain of varying length (C_12_-C_19_) [2]. As a biosurfactant, a concentration of 20 mM surfactin enables also the reduction of surface tension at water-air interfaces from 72 mN/m to 27 mN/m [3].

Surfactin is synthesized by a multi-modularly organized non-ribosomal peptide synthetase (NRPS) encoded by the tetracistronic *srfA* operon. NRPS gene expression is controlled by cell differentiation processes and in particular by the ComX-mediated quorum sensing mechanism, which enables biomass-related surfactin production [4–6]. In addition, the NRPS needs to be post-translationally activated by the 4-phosphopantetheinyl transferase Sfp [7]. Accordingly, in the well-established laboratory *B. subtilis* strain 168, its inactive *sfp* version needs to be genetically repaired to restore surfactin production [8]. In comparison, surfactin-producing derivatives of the non-sporulating *B. subtilis* strain 3NA (encoding a functional *sfp* gene version), a hybrid strain from *B. subtilis* 168 and W23 [9], showed improved surfactin production yields compared to strain JABs24, a surfactin-producing derivative of the *B. subtilis* strain 168, in both shake flask cultures and bioreactor fermentations [5, 10, 11]. While the two strains BMV9 and JABs32 show a knockout of the *manPA* operon for mannose phosphoenolpyruvate-dependent genome engineering system for development of markerless gene deletions (BMV9 shows a markerless *manPA* deletion and JABs32 a *manPA*::*erm*^*R*^ substitution), both surfactin-producing derivatives of strains 168 and 3NA have a native promoter region for the expression of the *srfA* operon [10, 11].

The main differences between JABs24 and the 3NA-derived strain JABs32 are a nonsense mutation in the *spo0A* gene, which leads to a lack of sporulation, and a C-terminal elongation of eleven amino acids in the *abrB* gene [9]. Both mutations are important for improved surfactin productivity in strain JABs32 [11]. As a result of the lack of sporulation, a surfactin-producing 3NA strain achieved a 1.6-fold higher growth rate in shake flasks, which makes this strain attractive for fermentation processes with high cell density [5, 11, 12]. In bioreactor cultures with optimized mineral salt medium, biomass concentrations of 41.3 g/L and surfactin titers of 23.7 g/L were achieved after 31 h of cultivation [5].

In alternative high cell density cultivations, different *B. subtilis* strains were used for production. For example, a *B. subtilis* strain from DMJ Biotech Corp. (Korea) was used for nattokinase production using pH-stat fed-batch culture, resulting in a nattokinase activity of 14,500 units/mL [13]. In addition, a yield of 3.16 g/L beef peptide was successfully produced using *B. subtilis* 168 with a self-inducible expression system containing a *srfA* promoter [14], while recombinant beta-galactosidase was produced in fed-batch culture in high cell density using *B. subtilis* 1S10 [15].

However, since not only the cell amount is relevant for the final yield, but also the cell productivity, the cultivation strategy within the bioprocess is of great importance. Regarding the production of biosurfactants by *B. subtilis* strain SPB1, Bouassida et al. [16] have shown that a fed-batch process is the more efficient strategy compared to batch cultivation. Furthermore, the authors compared different glucose feeding strategies, namely pulsed, constant and exponential feeding, which led to the conclusion that the highest concentration of 400 mg/L biosurfactants was achieved with exponential feeding [16].

In comparison, the *B. subtilis* wild-type strain BDCC-TUSA-3 was grown in a fed-batch bioreactor with an exponential feeding rate of about 0.435 h^− 1^ using Maldex-15, a by-product during manufacturing of high fructose syrup from corn starch, as substrate, which led to a maximum crude biosurfactant titre of 36 g/L (quantification after acetic precipitation and re-crystallization) and production yields Y_P/X_ of 1.13 g/g and Y_P/S_ of 0.272 g/g [17]. In the study of Willenbacher et al. [18], the *B. subtilis* wild-type strain DSM10T was utilized for surfactin production using a fed-batch cultivation with mineral salt medium and a single addition of glucose after passing through the batch phase, leading to a maximum surfactin titre of 1.22 g/L and production yields Y_P/X_ of 0.26 g/g and Y_P/S_ of 0.05 g/g [18]. In the study of Mei et al. [19], the effect of EDTA-Fe^2+^ on surfactin production with *B. subtilis* production strain ATCC 21332 could be shown in mineral salt medium. In subsequent sequential fed-batch fermentation processes, maximum surfactin titres of up to 9.41 g/L with specific productivities q_P/X_ of up to 0.056 g/g*h could be achieved [19]. Guo et al. [20] described the genetic modification of a surfactin-producing *B. subtilis* strain and applied a fed-batch fermentation with a feed solution containing 240 g/L glucose, 30 g/L tryptone, and 25 g/L beef extract as carbon sources, which was added automatically to maintain the dissolved oxygen at 40–50%. Using the genetically modified *B. subtilis* strain BSSF64, the authors were able to produce a surfactin titre of 3.89 g/L with a production yield Y_P/X_ of 0.63 g/g [20].

In this study, to address the question of the influence of growth rate on surfactin production in *B. subtilis* grown in a high cell density fed-batch process, a range of growth rates, namely 0.075, 0.15, 0.2, 0.25, 0.3 and 0.4 h^− 1^, were applied. Based on the obtained production performances achieved with strain *B. subtilis* BMV9, a surfactin-producing derivative of strain 3NA, correlations between the growth rate and the maximum surfactin titre, the production yields Y_P/X_ and Y_P/S_ as well as the productivities q_P/X_ and q_P/S_ could be calculated.

## Methods

### Chemicals and standards

Chemicals used in this study, if not otherwise stated, were purchased from Carl Roth GmbH & Co. KG (Karlsruhe, Germany). Surfactin standards (≥ 98% purity) were obtained from Sigma-Aldrich Laborchemikalien GmbH (Seelze, Germany).

### Bacterial strain, media and conditions for fed-batch cultures

The *Bacillus subtilis* strain BMV9 (*spo0A3*; *trp*^+^; *sfp*^+^; Δ*manPA*) was used in this study [5, 10]. In comparison to Klausmann et al. [5], the only difference to JABs32 is a removal of the *erm*^*R*^ resistance cassette from the *manPA* knockout region in BMV9. The media compositions used for precultures or fermentation processes were previously described by Klausmann et al. [5]. In brief, the first preculture was performed in LB-medium, while a chemically defined mineral salt medium was used for the subsequent second preculture as well as the final bioreactor fermentation culture [5].

The shake flask cultivations were carried out in an incubator shaker (NewbrunswickTM/Innova 44, Eppendorf AG, Hamburg, Germany) at 37 °C and 120 rpm. The bioreactor cultures were performed in a 30 L fermenter (ZETA GmbH, Graz/Lieboch, Austria) filled with 12 L batch medium. For protection against overfoaming, the bioreactor was connected with a foam trap described previously by Klausmann et al. [5]. The following parameters were set to a temperature of 37 °C, a pH value of 7.0 and an initial stirrer speed of 300 rpm using three Rushton turbines. Dissolved oxygen was adjusted to a minimum of 50% by adjusting the stirrer speed and aeration rate. After inoculation of 12 L of batch medium to an initial OD_600_ of 0.1, the cells were cultured at constant parameters of 37 °C, a pH of 7 and an aeration rate of 10 L/min until glucose was depleted as the sole carbon source. The associated cellular adaptation of metabolism for consumption of acetate as an alternative carbon source produced in non-affecting concentrations during the batch phase led to a slower metabolic rate and a characteristic increase of pO_2_. In this way, a real-time measurement was available for the identification of the feeding start within 1 min before the cell suspension entered the transient and stationary phase. This characterization and identification of the feeding start was previously described and experimentally established by Henkel et al. [21]. Afterwards, the initial aeration rate of 10 L/min was adjusted stepwise from 15 to 72 L/min when a 50% (w/w) glucose solution was added. The details of the cultivation process in both shake flask and one-step bioreactor fermentation have been described by Klausmann et al. [5]. One difference to be mentioned is the fact that instead of an additional supply of ammonia as nitrogen source, which was initially provided at 1 g/L in the batch medium, the further addition of ammonium was ensured via pH control by adding 20% (v/v) NH_3_ solution, which was maintained at a constant level of around 1 g/L ammonium (Figure S1).

The initial feeding rate F_0_ for the glucose feed was calculated directly after the batch phase according to the formula below. The initial feeding rate was used to calculate the feed rate F(t) at every time point (t) of the fed-batch.

1 $$\:{F}_{0}=\left(\frac{\mu\:}{{Y}_{X/S}}+m\right)*\left(\frac{{C}_{X,Batch}\:*\:{V}_{0}}{{C}_{S,Feed}}\right)$$

2 $$\:F\left(t\right)\:=\:{F}_{0}\:*\:{e}^{\mu\:\:*\:\:t}$$

In these equations, F_0_ is the initial feed rate (kg/h); F(t) the exponential feed rate at every time point t (kg/h); µ the targeted growth rate set to 0.075, 0.15, 0.2, 0.25, 0.3 or 0.4 h^− 1^; m the maintenance coefficient set to 0.05 g/(g*h); Y_X/S_ the conversion yield of glucose to biomass in the batch phase (g/g); C_X, Batch_ the biomass concentration at feed start (g/L); V_0_ the bioreactor volume in the batch (L) and C_S, Feed_ the glucose concentration in the feed (g/L).

### Sample analysis

The samples taken during cultivation were centrifuged at 3890 g for 10 min at 4 °C (Multifuge X3R, Thermo Fisher Scientific, Waltham, USA). The cell-free supernatants were used to quantify glucose with an enzymatic assay kit (R-Biopharm AG, Darmstadt, Germany), acetate with an enzymatic assay kit (R-Biopharm AG, Darmstadt, Germany) and ammonium with a photometric assay kit (Merck KGaA, Darmstadt, Germany). For the calculation of cell dry weight (CDW), samples (10 mL) from bioreactor approaches were taken. Cells were separated by centrifugation at 3890 g for 10 min at 4 °C. After washing in 9% (w/v) NaCl solution, cell pellets were dried at 110 °C for 24 h (memmert UF 110, Memmbert GmbH & Co.KG, Schwabach, Germany). After analysing 11 representative bioreactor samples from the fed-batch process, a mean correlation factor of 0.232 was calculated between CDW and the experimentally determined OD_600_ values.

### Surfactin quantification

Surfactin was only measured from the cultivation broth. Therefore, surfactin produced was quantified using high-performance thin-layer chromatography (HPTLC) (CAMAG AG, Muttenz, Switzerland). All experimental details were described by Geissler et al. [22]. In brief, 2 mL of the cell-free supernatant was used for a threefold extraction with chloroform/methanol (2:1). The pooled solvent layers were dried using a rotary evaporator (RVC 2–25 Cdplus, Martin Christ Gefriertrocknungsanlagen GmbH, Osterode am Harz, Germany) at 40 °C and 10 mbar. After dissolving in 2 mL methanol, the samples were applied in 6-mm bands to a silica HPTLC plate. Migration at a distance of 60 mm was performed with a mixture of chloroform/methanol/water (65:25:4) as mobile phase before surfactin detection at 195 nm [22]. A surfactin standard (Sigma Aldrich, Seelze, Germany) was used for quantification.

### Data analysis

All experiments were carried out in biological duplicates. The yield of product per biomass (Y_P/X_), product per substrate (Y_P/S_), biomass per substrate (Y_X/S_), specific productivity (q_P/X_) and specific substrate-product conversion rate (q_P/S_) were calculated for the feeding phase, excluding parameters from the batch phase, with the equations below. For these calculations, the parameters time (t), biomass (X), glucose as substrate (S) and surfactin as product (P) were used. More specifically, the time point of maximum surfactin concentration was used to determine the Y_P/S_ and Y_P/X_ values, while Y_X/S_ was determined at time point of maximum biomass formation.

3 $$\:{Y}_{P/X}=\:{\left.\frac{P}{X}\right|}_{P={P}_{max}}$$

4 $$\:{Y}_{P/S}=\:{\left.\frac{P}{S}\right|}_{P={P}_{max}}$$

5 $$\:{Y}_{X/S}=\:{\left.\frac{X}{S}\right|}_{X={X}_{max}}$$

6 $$\:{q}_{P/X}=\:\frac{{P}_{max}}{X\:*\:t}$$

7 $$\:{q}_{P/S}=\:\frac{{P}_{max}}{S\:*\:t}$$

### Fitting of experimental data and production parameters

All graphs were generated using OriginPro 2022b (OriginLab Corporation, Northampton, United States) software. The data series for acetate, ammonium, CDW, glucose, and surfactin were analyzed utilizing polynomial functions. The production parameters were analyzed using the Simple Fit tool with existing functional equations (polynomial or exponential) to maximize the coefficient of determination.

## Results

### Description of comparative bioreactor batch phases

**Fig. 1:**
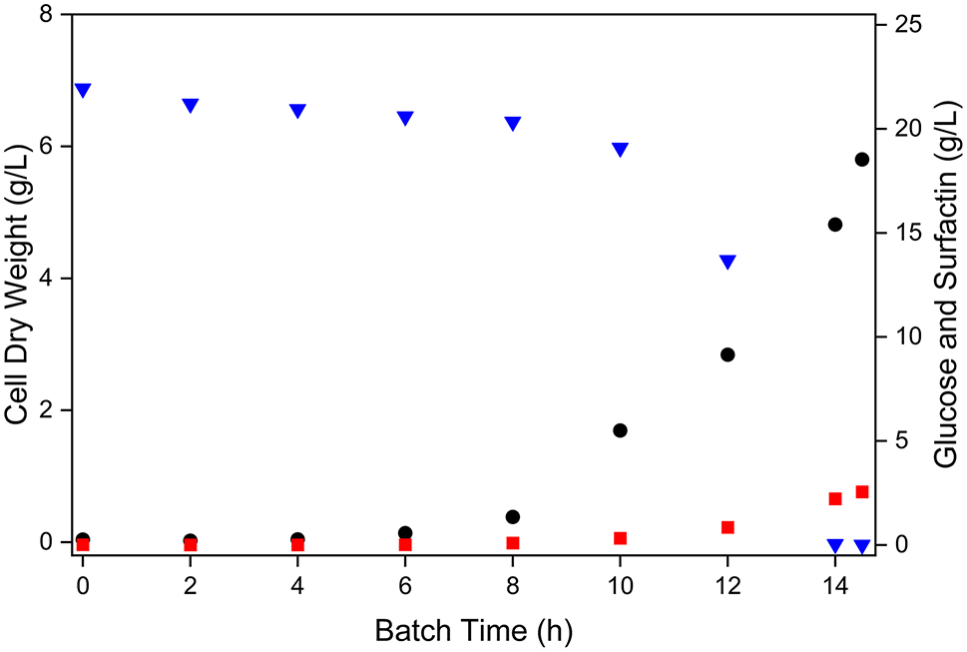
Representative batch cultivation procedure using *B. subtilis* BMV9. The figure shows the time-dependent kinetics of cell dry weight (black cycles), glucose availability (blue inverted triangles) and surfactin production (red squares) during the cultivation process

After inoculation of 12 L of batch medium, the batch phase of each bioreactor cultivation process was performed as described previously [5]. The initial amount of glucose (25 g/L) was metabolised between 11.5 and 16 h, resulting in a mean CDW value of 7.22 ± 0.82 g/L and a mean surfactin concentration of 3.87 ± 0.65 g/L. During this cultivation process, an accumulation of up to 0.57 g/L acetate was detected as an overflow by-product (Figure S2A). A representative batch phase for the cultivation of *B. subtilis* BMV9 is shown in Fig. 1 before different feeding rates were set for the subsequent cultivation process.

### Effects of the feeding rates on surfactin production

After glucose depletion, the respective feeding processes were started at different exponential rates until a final maximum bioreactor volume of approximately 19 L was reached. Accordingly, a lower growth rate led to a lower glucose availability and a prolonged cultivation time of the feeding process. Consequently, at a growth rate of 0.075 h^− 1^ a cultivation time of about 27.5 h after the start of feeding was required, at 0.15 h^− 1^ about 15 h, at 0.2 h^− 1^ about 11 h, at 0.25 h^− 1^ about 9 h, at 0.3 h^− 1^ about 7 h and at 0.4 h^− 1^ about 5 h. Nevertheless, comparable CDW values of approximately 42.9 ± 5.1 g/L were achieved at the end of each bioreactor process. More specifically, the highest CDW of 48 ± 0.9 g/L was achieved at a growth rate of 0.25 h^− 1^, while lower biomass formation was observed at higher growth rates of 0.3 h^− 1^ (46.4 ± 1.2 g/L) and 0.4 h^− 1^ (36.3 ± 0.4 g/L). As a result, glucose (11.6 ± 0.2 g/L) accumulated during the 0.3 h^− 1^ feeding process until 5.5 h after the start of feeding, before the remaining glucose was metabolised. In contrast, a continuous glucose accumulation of up to 48.5 g/L ± 4.3 g/L was detectable at the growth rate of 0.4 h^− 1^. In this context, a production of acetate associated with overflow metabolism was detected mainly at these growth rates (2.6 ± 0.3 g/L at 0.3 h^− 1^ and 1.8 ± 1.2 g/L at 0.4 h^− 1^, Table S1 and Figure S2). Nevertheless, the conversion of glucose as substrate to biomass Y_X/S_ could be determined with nearly constant values for growth rates higher than 0.15 h^− 1^ during the feeding process, suggesting a higher impact of maintenance during the growth rates of 0.075 and 0.15 h^− 1^.

**Fig. 2:**
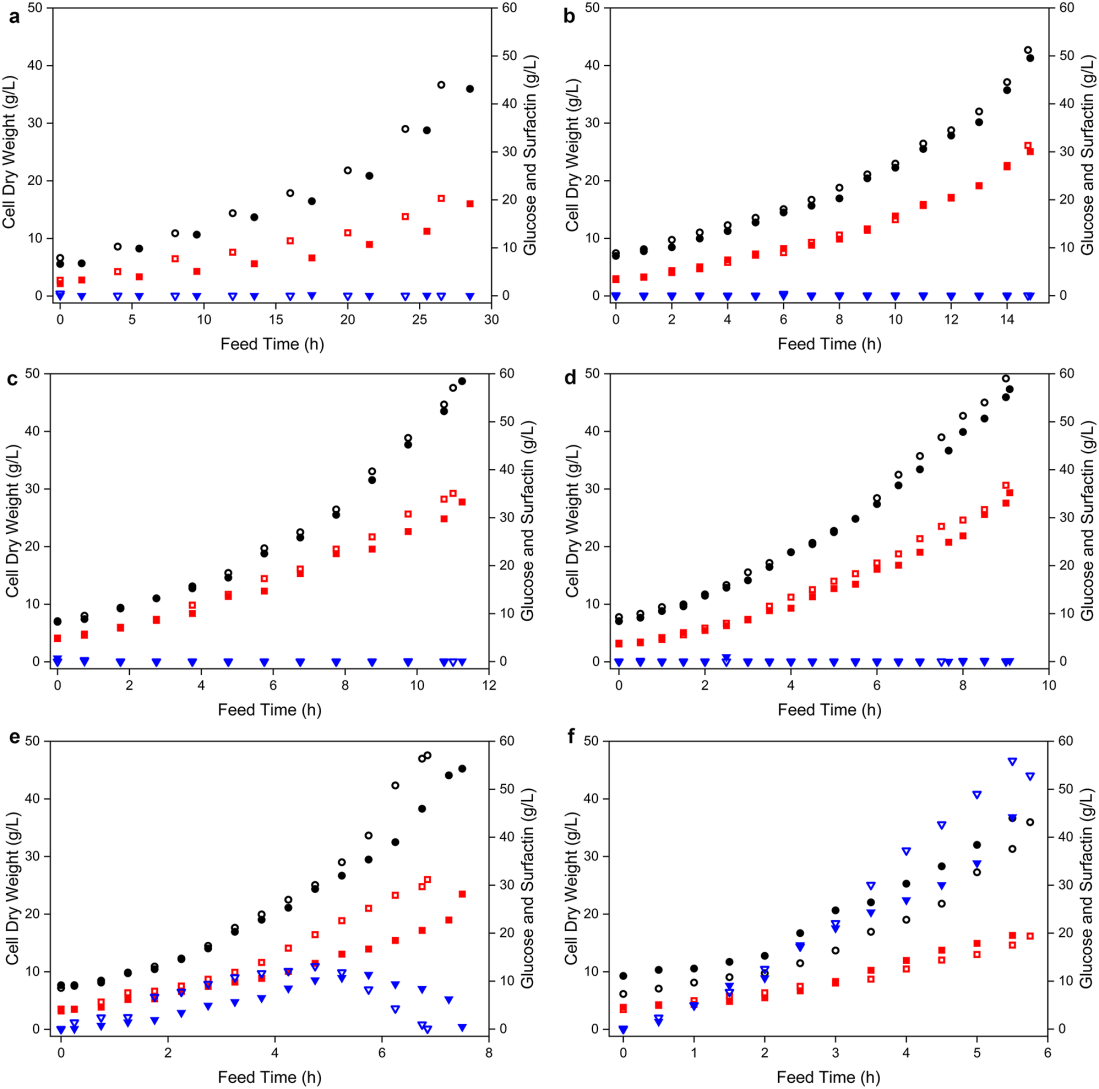
Time course of bioreactor cultivations of *B. subtilis* BMV9 with different growth rates for the production of surfactin. After depletion of glucose in the batch phase, the feed solution was added at different growth rates of 0.075 h^− 1^ (**A**), 0.15 h^− 1^ (**B**), 0.2 h^− 1^ (**C**), 0.25 h^− 1^ (**D**), 0.3 h^− 1^ (**E**) and 0.4 h^− 1^ (**F**). The fed-batch process was stopped when a feeding volume of 6 L was depleted. The process parameters used were cell dry weight (black circles), glucose (blue inverted triangles) and surfactin concentration (red squares). Each feeding rate used for the fed-batch bioreactor process was performed in biological duplicates

Overall, the production of surfactin followed the trend of biomass formation. Thus, the highest surfactin concentration of 36.0 ± 0.8 g/L was measured at a growth rate of 0.25 h^− 1^, while concentrations of 19.7 ± 0.6 g/L, 30.7 ± 0.6 g/L and 34.2 ± 0.9 g/L were observed at 0.075 h^− 1^, 0.15 h^− 1^ and 0.2 h^− 1^, respectively. In addition, the lower biomass formation observed at the growth rates of 0.3 h^− 1^ and 0.4 h^− 1^ also resulted in lower surfactin concentrations of 29.7 ± 1.5 g/L and 19.4 ± 0.1 g/L, respectively. A complete overview of the growth behaviour and production kinetics at the different feeding rates of the fed-batch cultivation processes is summarized in Fig. 2.

Overall, a correlation between biomass formation and surfactin production was observed. In addition, the cultivation parameters were used to calculate production yields and productivities as shown in Table 1. Accordingly, the most effective conversion of glucose as the sole carbon source into surfactin (Y_P/S_) was determined at growth rates of 0.2 and 0.25 h^− 1^, while comparatively low or high growth rates of 0.075 h^− 1^ and 0.4 h^− 1^, respectively, resulted in significantly lower production yields. In terms of biomass productivity (Y_P/X_), the highest values were calculated for feeding rates of 0.15 h^− 1^, 0.2 h^− 1^ and 0.25 h^− 1^. However, when looking at the specific productivities q_P/S_ and q_P/X_, growth rates of 0.25 h^− 1^ and higher show the best values. Finally, the highest maximum surfactin titres were achieved with a moderate feeding rate of 0.25 h^− 1^.

**Table 1:** Evaluation of the production yields and specific productivities for the surfactin production using different feeding rates. All values were calculated in the fed-batch phase

	0.075 h^-1^	0.15 h^-1^	0.2 h^-1^	0.25 h^-1^	0.3 h^-1^	0.4 h^-1^
Max. titre [g/L]	19.7	30.7	34.2	36.0	29.7	19.5
Y_P/S_ [g/g]	0.11	0.18	0.20	0.21	0.17	0.15
Y_P/X_ [g/g]	0.47	0.68	0.65	0.70	0.59	0.46
Y_X/S_ [g/g]	0.20	0.23	0.28	0.27	0.26	0.28
q_P/S_ [g/(g*h]	0.0038	0.012	0.018	0.023	0.024	0.027
q_P/X_ [g/(g*h)]	0.017	0.046	0.058	0.077	0.082	0.082

### Correlation between growth rate, surfactin production and specific productivities

**Fig. 3:**
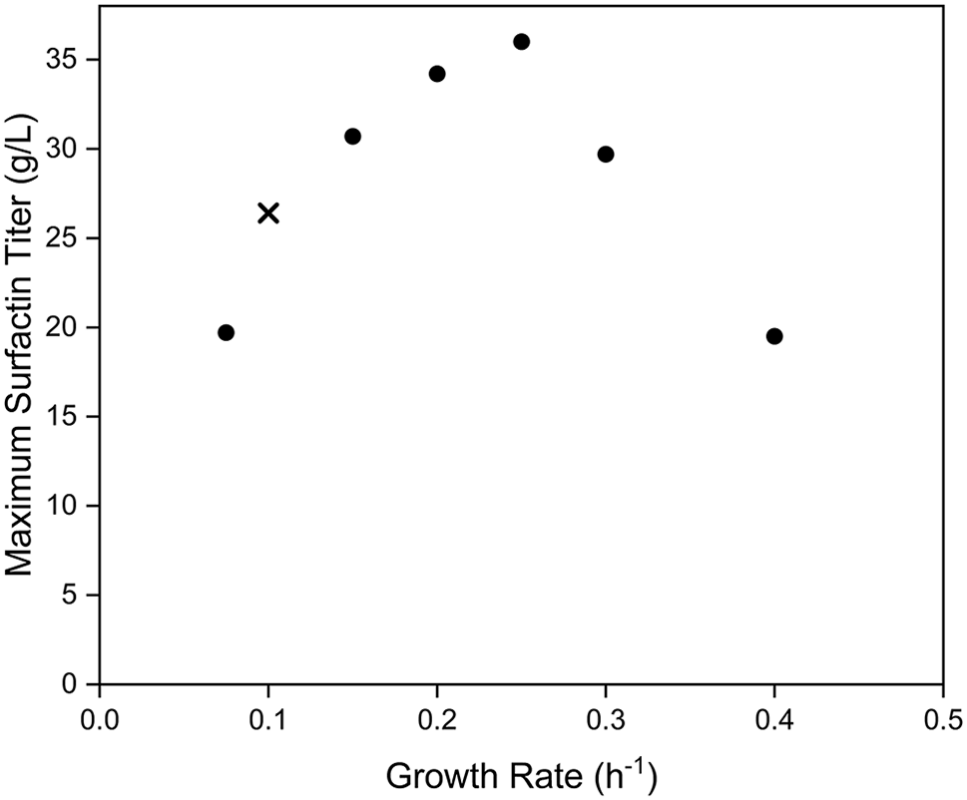
Correlation between growth rate and maximum surfactin titre. Six different growth rates during the feeding phase of the bioreactor experiments in the range of 0.075 h^− 1^ and 0.4 h^− 1^ (circles) were determined. In addition, reference results from Klausmann et al. [5] with a growth rate of 0.1 h^− 1^ (cross) were integrated

The dependency between surfactin production and adjusted growth rate during the feeding phase of the bioreactor cultivation processes reveals maximum surfactin titres produced during the fed-batch approach with feeding rates between 0.2 and 0.25 h^− 1^. To prove the overall correlation, results published previously by Klausmann et al. [5] were also integrated into the correlation shown in Fig. 3. In this study, a comparable bioreactor approach was performed for surfactin production using the sporulation-deficient strain *B. subtilis* JABs32, a derivative of the BMV9 strain.

Overall, using the described bioreactor cultivation parameters, a feeding rate of about 0.25 h^− 1^ seems to allow the highest maximum surfactin titre of about 36 g/L, while more unproductive biomass was observed at lower growth rate settings and an additional effect due to stimulation of overflow metabolism, as shown for acetate production, seems to reduce surfactin production at higher feeding rates. Comparable kinetics could also be shown for the correlation between the adjusted growth rates and the yields Y_P/X_ and Y_P/S_ calculated for surfactin production (Figure S3). Accordingly, the adjusted feeding rate and the correlated growth rate have a significant influence on the efficiency of surfactin production in the described bioreactor concept.

**Fig. 4:**
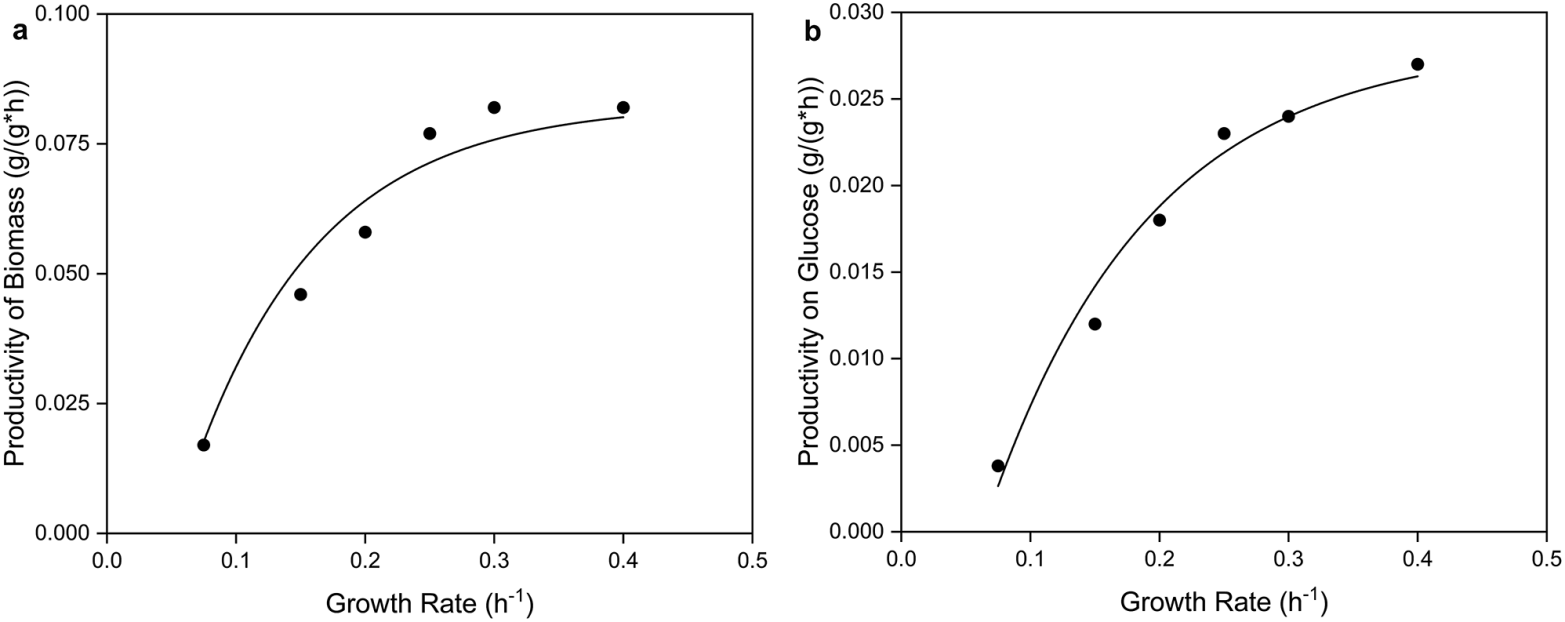
Coherence between growth rate and specific surfactin productivities. The specific productivity of biomass (**A**) and the specific productivity on glucose (**B**) show a clear correlation to the growth rate with a trend towards higher productivities at higher growth rates, but ending in a saturation after 0.25 h^− 1^. Based on the correlation between the adjusted growth rate during the feeding phase and the specific surfactin productivities, exponential equations for biomass and glucose could be derived

In contrast, the correlations between the growth rate obtained in the feed phase of the fed-batch bioreactor process and the specific productivities for biomass (q_P/X_) and substrate-product conversion rate (q_P/S_) show a trend towards higher productivities at higher growth rates. However, a saturation of the increase was clearly observed at 0.25 h^− 1^, indicating a more inefficient surfactin production at higher glucose availability, as already described for an additional production of overflow metabolites (Fig. 4). Accordingly, the higher the growth rate, the less the specific productivity increases.

Nevertheless, the correlations allow the derivation of functional equations for the specific productivities in terms of surfactin production in the fed-batch bioreactor cultivation approaches described:

8 $$\begin{aligned}q(P/X)\,=&\,-0.11 (g_P/(g_X *h)) \\&*(-1.25 *(1 -exp(-10  (1/h) * \mu))+0.5)\end{aligned}$$

9 $$\begin{aligned}q(P/S)\,=&\,-0.037 (g_P/(g_S *h)) \\& *(-1.25 *(1 -exp(-8 (1/h) * \mu))-0.39)\end{aligned}$$

The functional equation generated by the Simple Fit tool describes the relationship between the specific surfactin productivities and growth rate as an exponential function with determination values R^2^ of 0.95 for Eq. (8) and 0.98 for Eq. (9). The prefactors were adjusted to include the appropriate units, ensuring that the result of the equation is expressed in g/(g*h). The functional equation is valid within the experimental growth rate range from 0.075 h^− 1^ to 0.4 h^− 1^.

## Discussion

In previous studies on surfactin production, *B. subtilis* strains with established sporulation deficiency have proven to be favourable production organisms [5, 11]. For this reason, *B. subtilis* BMV9 was used as a promising surfactin production strain [10]. However, the challenge remained to identify the most productive surfactin production conditions during fed-batch bioreactor operation. To address this issue, fed-batch bioreactor processes were established according to previous work from Klausmann et al. [5], with a constant amount of ammonium of around 1 g/L being ensured by adding a 20% (v/v) NH_3_ solution for pH control (Figure S1).

In comparative fed-batch bioreactor processes, the only variable parameter introduced was a different growth rate (0.075, 0.15, 0.2, 0.25, 0.3, 0.4 h^− 1^) after the respective batch phase in order to ensure different growth rates of the surfactin-producing *B. subtilis* BMV9 strain. In this way, the goal was to be able to predict under which nutrient availability in terms of glucose as the only carbon source, the most favourable substrate conversion to the target product surfactin can be achieved and thus to be able to develop functional equations in order to make predictions for varying feeding rates under comparable bioreactor processes and surfactin-producing strains. Furthermore, an increased feeding rate with the same maximum bioreactor filling volume and, ideally, increased productivity allows for a reduction in process time with a higher overall yield. This was the case by comparing the results in this study using a growth rate of 0.25 h^− 1^ and the results from Klausmann et al. [5] with a growth rate of 0.1 h^− 1^.

As a result of the comparative fed-batch bioreactor experiments, it was found that a growth rate of up to 0.25 h^− 1^ led to immediate metabolization of glucose as a carbon source, as a consistently low amount of glucose could be measured after feed start, whereas increased glucose accumulation was observed at 0.3 and 0.4 h^− 1^ (Fig. 2). Consequently, the cells were not able to utilise such a high amount of glucose per time and thus a maximum metabolism rate between 0.25 and 0.3 h^− 1^ could be identified. As a consequence, acetate accumulation was detected due to the stimulation of overflow metabolism at these feeding rates (Figure S2) [23, 24]. Since increasing acetate formation has a negative effect on the cell growth of *B. subtilis* [25], the final CDW was lower at the higher growth rates of 0.3 and 0.4 h^− 1^, resulting in a lower amount of productive biomass, which also produced overflow metabolites as additional by-products. This outcome is in contrast to the results of Amin [17], describing that higher growth rates lead to steadily increasing maximum surfactin titres of up to 36 g/L with a growth rate of 0.435 h^− 1^, associated with constant Y_P/X_ and Y_P/S_ values and increasing productivities [17]. However, this study worked with *B. subtilis* strain BDCC-TUSA-3, a wild-type isolate from solid waste, cultured at 30 °C in maldex-15, a by-product recovered during manufacturing of fructose syrup from corn starch, as a complex substrate. Similar observations were done for the bioproduction of beta-galactosidase in fed-batch cultivations with a mineral medium and glucose as substrate, leading to a highest yield at the highest growth rate of 0.38 h^− 1^ using the BB804 strain [26].

In contrast, a low growth rate, such as 0.075 h^− 1^, only appeared to provide nutrients for biological maintenance, leading to less productive biomass. Accordingly, the impact of maintenance appears to be more dominant the lower the feeding rate, resulting in reduced surfactin productivities in much lower growth rates. In contrast, the most effective product-forming biomass was identified at a growth rate of 0.25 h^− 1^, meaning under conditions where a high glucose availability was provided but no accumulation was detectable after feed start (Y_P/X_ = 0.7 g/g), which resulted in a final maximum surfactin titre of 36 ± 0.8 g/L. (Fig. 3). The correlations between the growth rates defined by the feeding rates and the yields Y_P/S_ and Y_P/X_ determined from the bioreactor experiments followed the kinetics already presented in Fig. 3 (Figure S3). In contrast, a maximum crude biosurfactant titre of 36.07 g/L was reported by Amin [17] with production yields Y_P/X_ of 1.13 g/g and Y_P/S_ of 0.272 g/g. However, a complex substrate, namely maldex-15, was used as substrate and an alternative quantitative surfactin precipitation was applied with acetic precipitation and re-crystallization [17]. In Bouassida et al. [16], the *B. subtilis* wild-type strain SPB1 was used in a fed-batch cultivation using shake flasks with exponential growth rate of 0.377 h^− 1^ with glucose as carbon source, leading to a maximum surfactin titre of 0.4 g/L and a production yield Y_P/X_ of 0.016 g/g, while the study from Mei et al. [19] described a maximum surfactin titre of up to 9.414 g/L with the *B. subtilis* production strain ATCC 21332 in serial fed-batch cultivations in shake flasks and adjusted EDTA-Fe^2+^ concentrations in a mineral medium with glucose as substrate, resulting in a specific productivity q_P/X_ of 0.056 g/g*h. In the study of Willenbacher et al. [18], the *B. subtilis* wild-type strain DSM10T was used for surfactin production in defined mineral salt medium and glucose as carbon source [18]. However, in contrast to the present work, a single addition of glucose was performed after a depletion of glucose at the end of the batch phase, which allowed a final surfactin titre of 1.22 g/L and production yields Y_P/X_ and Y_P/S_ of 0.26 and 0.05 g/g, respectively [18]. In contrast, a maximum surfactin titre of 3.89 g/L was reached with the genetically modified *B. subtilis* strain BSSF64, a derivative of the domesticated laboratory *B. subtilis* strain 168, using a mineral salt medium supplemented with tryptone and beef extract and a subsequent automatically addition of feed solution containing glucose, tryptone, beef extract, L-leucine and FeSO_4_ · 7 H_2_O to maintain the dissolved oxygen at 40 − 50%. In this way, a surfactin production yield Y_P/X_ of 0.63 g/g was reached in a 5 L-bioreactor [20].

In addition to the overall surfactin production, the correlations between the specific time-dependent productivity q_P/S_ or q_P/X_ and the growth rates were also compared in this present study. Here, an increase in the specific productivities with increasing growth rate was shown, although a saturation in the increase was observed from a growth rate of approx. 0.25 h^− 1^ (Fig. 4). Accordingly, further increases in both q_P/S_ and q_P/X_ are associated with significantly higher growth rates, which makes the efficiency of higher feed rates less favourable. At this point, the conclusion to be drawn is that the time saved is not in proportion to the reduced productivity of the biomass and the final amount of surfactin formed.

The correlation between substrate-product conversion and growth rate could also be demonstrated and thus the most efficient surfactin production could be determined with constant process control and the growth rate as a varying parameter, while the total biomass remained constant in all bioprocesses. Consequently, the influence of the growth rate on the efficiency of surfactin production could be simulated. Further studies should also address which molecular regulatory networks contribute to surfactin production during a growth rate of 0.25 h^− 1^ compared to less efficient growth rates such as 0.075 h^− 1^.

## Conclusion

The control of the growth rate during a bioprocess has a crucial impact on the production capacity of the respective production organism. In the case of the sporulation-deficient surfactin-producing strain *B. subtilis* BMV9, a growth rate of 0.25 h^− 1^ proved to be particularly efficient, whereas higher feeding rates led to an activation of overflow metabolism and lower glucose availabilities only appeared to serve the fundamental metabolism. In addition to ensuring more productive biomass, an efficient fed-batch bioprocess control could also result in a reduction in process time, which needs to be determined individually for the production strain and the target substance to be produced.

## Electronic supplementary material

Below is the link to the electronic supplementary material.

Supplementary Material 1

Supplementary Material 2

## Data Availability

No datasets were generated or analysed during the current study.
