# Supplementary material for: The influence of growth rate-controlling feeding strategy on the surfactin production in Bacillus subtilis bioreactor processes
Source: Microb Cell Fact. 2024 Sep 30;23:260. doi: 10.1186/s12934-024-02531-w (PMC11440882; doi:10.1186/s12934-024-02531-w)
Supplement: Supplementary file 1 — Supplementary Material 1 [file 12934_2024_2531_MOESM1_ESM.docx]

**The influence of growth rate-controlling feeding strategy on the surfactin bioproduction in *Bacillus subtilis* bioreactor processes**

Eric Hiller^1^ (ORCID ID: 0009-0000-3815-920X), Manuel Off^1^(ORCID ID: 0009-0009-3585-6822), Alexander Hermann^1^ (ORCID ID: 0009-0000-5431-0723), Maliheh Vahidinasab^1^ (ORCID ID: 0000-0002-0660-787X), Elvio Henrique Benatto Perino^1*^ (ORCID ID: 0000-0003-0372-260X), Lars Lilge^1*^ (ORCID ID: 0000-0002-7693-477X), Rudolf Hausmann^1^ (ORCID ID: 0000-0002-2327-7120)

^1^ Department of Bioprocess Engineering, Institute of Food Science and Biotechnology, University of Hohenheim, Stuttgart, Germany.

*Corresponding author: [lars.lilge@uni-hohenheim.de](mailto:lars.lilge@uni-hohenheim.de) and eperino@uni-hohenheim.de





**Figure S1. Time course of the ammonium concentration during the feeding phase of fed-batch bioreactor processes.** Shown are the ammonium concentrations of two independent fed-batch bioreactor experiments (filled and empty squares), which were maintained by pH control over time.







**A B**

**Figure S2. Actetate profile during the surfactin production processes.** The accumulation of acetate was measured during the batch phase (A) as well as during the different feeding phases (B). High growth rates such as 0.4 1/h (blue squares) favoured acetate accumulation, while 0.25 1/h (black squares) and lower growth rates showed a nearly undetectable acetate concentration over the feeding time.

**

**

**A B**

**

**

**C**

**Figure S3. Correlation between growth rate and yields.** The yields for glucose-surfactin conversion Y_P/S_ (A), surfactin production yield by the biomass Y_P/X_ (B) and glucose to biomass conversion Y_X/S_ (C) were plotted as a function of the respective growth rates set by the feeding rates. A fitting curve was applied to both correlations in order to work out the dependencies of the yields on the defined growth rates.
